# Teacher-Evaluated Self-Regulation Is Related to School Achievement and Influenced by Parental Education in Schoolchildren Aged 8–12: A Case–Control Study

**DOI:** 10.3389/fpsyg.2018.00438

**Published:** 2018-04-04

**Authors:** Marleen A. J. van Tetering, Renate H. M. de Groot, Jelle Jolles

**Affiliations:** ^1^Faculty of Behavioral and Movement Sciences, Centre for Brain & Learning, Vrije Universiteit Amsterdam, Amsterdam, Netherlands; ^2^Welten Institute, Research Centre for Learning, Teaching, and Technology, Open University of the Netherlands, Heerlen, Netherlands; ^3^NUTRIM School of Nutrition and Translational Research in Metabolism, Maastricht University, Maastricht, Netherlands

**Keywords:** self-regulation, school achievement, late childhood, early adolescence, parental education, executive functions

## Abstract

There are major inter-individual differences in the school achievements of students aged 8–12. The determinants of these differences are not known. This paper investigates two possible factors: the self-regulation of the student and the educational levels obtained by their parents. The study first investigates whether children with high and low academic achievement differ in their self-regulation. It then evaluates whether there are differences in the self-regulation of children with high and moderate-to-low level of parental education (LPE). The focus was on the self-regulation of students as judged by their teacher. Teacher evaluations were assessed using an observer questionnaire: the Amsterdam Executive Functioning Inventory. Results showed that students with low school achievement had substantially lower teacher-perceived self-regulation than children with high school achievement. Furthermore, teacher-perceived self-regulation was lower for children with moderate-to-low LPE than for children with high LPE. The findings suggest that interventions on the domain of self-regulation skills should be developed and used, particularly in students at risk of poor school achievement.

## Introduction

Children in the age-periods of late childhood and early adolescence (i.e., aged 8–12) are characterized by major inter-individual differences in school achievement and learning performance (e.g., Gerst et al., 2015). Skills in the domain of self-regulation may have a key role in determining differences in school achievement (e.g., Clark et al., 2010; Arrington et al., 2014; Cragg and Gilmore, 2014; Dekker et al., 2017). Self-regulation includes abilities such as concentrating on tasks for longer periods of time, suppressing impulsive behavior, planning the smaller steps that are necessary to solve tasks, planning future activities, and prioritizing tasks (Anderson, 2002; Lezak et al., 2012; Diamond, 2013; Chen et al., 2015; Gerst et al., 2015; Jolles, 2016). These abilities are considered to be important for school achievement (Anderson, 2002; Bembenutty et al., 2013; Diamond, 2013; Gerst et al., 2015). Inter-individual variations in the pace at which these abilities develop could therefore contribute to differences in the school performances of students. Another factor which has been mentioned as a potential determinant of inter-individual differences in the pace at which cognitive abilities develop is the level of parental education (LPE) (Ardila et al., 2005; van Tetering and Jolles, 2017). This study therefore aimed to investigate the importance of self-regulation for academic achievement while taking the LPE into consideration.

Self-regulation has been conceptualized in various ways and a word about its definition is therefore warranted. Self-regulation involves modulating systems of emotion, attention, and behavior in response to a given situation or stimulus (Carlson, 2003; Eisenberg et al., 2004; Ferrier et al., 2014). This includes managing emotions, shifting or focusing attention, and both inhibiting and activating behaviors (Smith-Donald et al., 2007). In the present study, we particularly focus on the ability to regulate one’s behaviors and thoughts in order to executive goal directed behavior. Executive functions sub serve the capacity to self-regulate (see Jahromi and Stifter, 2008; Hofmann et al., 2012; Nigg, 2017). Executive functions include a set of higher-order abilities such as working memory, mental flexibility, planning, prioritizing, impulse regulation, assessing the consequences of actions, and attentional functions. These abilities provide the cognitive foundation for self-regulation (see Hofmann et al., 2012; Diamond, 2013; Gunzenhauser et al., 2017). Complex brain networks – including those which connect the prefrontal cortex to other cortical and subcortical brain areas – have been associated with these self-regulatory skills (Giedd and Rapoport, 2010; Lenroot and Giedd, 2011; Leshem, 2016). The development of self-regulation matches the maturational stages of these brain areas and networks (Huizinga et al., 2006). Both improve progressively at a linear pace during childhood and adolescence, before plateauing upon reaching adulthood (Anderson, 2002; Harden and Tucker-Drob, 2011; Diamond, 2013; Steinberg et al., 2017).

Several studies suggest that factors such as LPE influence a child’s cognitive development (Ganzach, 2000; Rosenzweig, 2003; Ardila et al., 2005; Sameroff, 2010; Noble et al., 2015; Rindermann and Baumeister, 2015), and may also affect the development of self-regulation. The LPE is the level of education that the child’s parents have obtained. Previous studies have used this measure as a proxy for the level of intellectual stimulation in the home environment (e.g., Hoff, 2003; Ardila et al., 2005; Rindermann and Baumeister, 2015). It appears that parents with higher levels of education create a more intellectually stimulating environment for their children than parents with lower levels of education (Hoff, 2003). For example, it has been demonstrated that well-educated parents have a different way of interacting with their children particularly with respect to the language used (Hoff et al., 2002). College-educated mothers talk more, use a richer vocabulary, and read more often to their children than mothers who have high school as their highest level of education (Hoff-Ginsberg et al., 1991). As a result, children of college-educated parents tend to have a larger vocabulary and a more rapid language development (Ganzach, 2000; Carr and Pike, 2012; Kautz et al., 2014). LPE has also positively been correlated with children’s school attendance and general cognitive development (e.g., Ganzach, 2000; Carr and Pike, 2012; Kautz et al., 2014). Earlier studies from our department showed differences between children with higher and lower LPE in their problem-solving behavior and attention (Hurks et al., 2006; Meijs et al., 2009). We also found associations with planning and initiative taking at the end of primary school as evaluated by teachers (van Tetering and Jolles, 2017). Interesting findings from neuroscientific research are congruent with these findings; children of less well-educated and well-educated parents show notable differences in hippocampal volumes (Noble et al., 2015). These findings imply that there are substantial differences in the cognitive development of children growing up in higher LPE families and children growing up in lower LPE families. LPE may therefore also affect the development of self-regulatory skills. To our knowledge, the evaluation of academic performance in relation to the self-regulation of children with high and moderate-to-low LPE has not received much research attention. This is therefore one of the aims of the present study.

Another aim of the study is to investigate the importance of self-regulation in school achievement. With respect to this notion, recent papers have been published which show the importance of self-regulation to mathematics, spelling and reading comprehension at primary school (e.g., Clark et al., 2010; Friso-van den Bos et al., 2013; Arrington et al., 2014; Cragg and Gilmore, 2014; Gerst et al., 2015; Dekker et al., 2017; Schwaighofer et al., 2017). Mathematics calls for self-regulation when the student has to focus on the task, while disregarding irrelevant information, and choosing the right computational methods (Gerst et al., 2015; Dekker et al., 2017). Better self-regulation enables the student to solve the task with a step-by-step computational method, to monitor the progression, and to choose a more appropriate method when needed (e.g., Raghubar et al., 2010; Friso-van den Bos et al., 2013; Yeniad et al., 2013; Cragg and Gilmore, 2014; Gerst et al., 2015; Dekker et al., 2017). Likewise, spelling requires self-regulation while efficiently integrating phonological, orthographical, and morphological information (Berninger et al., 2006; Garcia et al., 2010; Dekker et al., 2017). Furthermore, reading comprehension requires children to focus on words and sentences that are relevant to the main topic and ignore additional information that is presented (Arrington et al., 2014; Gerst et al., 2015). Moreover, previous studies reported that the student’s ability to process new information and to develop learning strategies is linked with their regulation of attention (e.g., McClelland et al., 2000; Blair, 2002; Howse et al., 2003; Zelazo et al., 2003; Fantuzzo et al., 2004; Smith-Donald et al., 2007). It is clear that optimal school performance requires high levels of self-regulation: children need to regulate their own attention, behavior, thoughts, and emotions in order to pay the required amount of attention to the learning tasks (Diamond, 2013). Differences in the self-regulation of students with higher and lower school achievement are therefore to be expected. To evaluate this notion was another aim of the present study.

In summary, our investigation focused on whether there are differences in the self-regulation of children from high and moderate-to-low LPE families (lower than vocational training vs. vocational training or higher), and whether there are differences in the self-regulation between children with higher and lower school achievement. A notable and important characteristic of the present study is the use of teacher evaluations to assess self-regulation. We chose this approach as teachers can be expected to have a better judgment of the students’ self-regulation skills than the students themselves because these skills and the students’ self-insight are still in development at the age of 8–12 (Diamond, 2013; Weil et al., 2013). Furthermore, teacher ratings of self-regulation can be considered highly ecologically valid (Kent et al., 2014; Chen et al., 2015; Dekker et al., 2017). This is because teachers have extensive contact with a given student, and offer valuable insight into the ways that students regulate their thoughts and behavior (Smith-Donald et al., 2007). Teachers in daily practice directly observe children during the performance of academic tasks within the school context, whenever planning, concentration, behavioral control, problem-solving, and the suppression of impulsive behavior are required (Diamond, 2013). Teachers can thus draw on multiple experiences and observations when rating their students’ self-regulatory actions and beliefs (Chen et al., 2015). Therefore, teacher-evaluated self-regulation could provide an appropriate estimation of the self-regulatory skills of children that are vital for academic achievement (Dekker et al., 2017). It is important to gain insight into the way teachers evaluate the self-regulatory skills of their students. Teacher-evaluations are an indication of their perception which determines the way they react to their students, and the way they intellectually stimulate their students (Summers et al., 2017). For instance, teachers frequently reported drawing on their beliefs about a child’s abilities when determining how to respond to children’s interaction (Summers et al., 2017). Teachers may give more challenging assignments to children if they believe that they are able to pay attention for a longer period of time and to plan their schoolwork properly. These high-level assignments stimulate cognitive development (Summers et al., 2017). Insight into the way teachers evaluate their students’ skills may therefore provide relevant new information about teacher-behaviors toward students.

Teacher-perceived self-regulation was assessed with a questionnaire which has been developed to evaluate abilities on the domain of cognitive control and self-regulation: the Amsterdam Executive Functioning Inventory (AEFI) (Van der Elst et al., 2012). The AEFI evaluates the three main aspects of self-regulation, namely (1) attention, (2) planning and initiative taking, and (3) self-control and self-monitoring. The AEFI previously showed its effectiveness in detecting age, sex, and LPE differences in teacher-perceived self-regulation (Van der Elst et al., 2012; van Batenburg-Eddes and Jolles, 2013; Baars et al., 2015; van Tetering and Jolles, 2017).

Another notable aspect of this study is the use of standardized national tests for the evaluation of school achievement: i.e., the so-called CITO tests (i.e., made by the Central Institute for Tests Development in the Netherlands). These tests are widely used by Dutch primary schools to monitor the learning progress of children in mathematics, reading, spelling, and reading comprehension from first to sixth grade. In this study, performances on three of these tests were used; mathematics (Janssen et al., 2010), spelling (de Wijs et al., 2010; Mols and Kamphuis, 2012), and reading comprehension achievement (Feenstra et al., 2010; Weekers et al., 2011). Earlier studies that investigated the link between self-regulation and school achievement used school grades as a proxy for school achievement (e.g., Cohen et al., 1995). However, school grades are known to suffer from variability in assessment and grading practices (Bowers, 2011; Reed et al., 2015). Other studies used achievement tests that are independent of actual school performances (e.g., Best et al., 2011; Arrington et al., 2014). A major strength of the CITO tests is that the majority of primary schools in the Netherlands use these instruments and good national norms are available. This makes the results of our study highly generalizable to everyday educational practice. We hypothesized that teachers would have a more negative evaluation of the self-regulation of children with moderate-to-low LPE than of children with high LPE. In addition, children with higher levels of school achievement can be expected to have higher teacher-evaluated self-regulation than children with lower levels of school achievement.

## Materials and Methods

### Participants and Procedures

The data used in this study were derived from a cross-sectional study into the determinants of learning performance and neurocognitive development during late childhood and early adolescence. This study involved *N* = 310 participants aged 8–12. The data were collected in April 2014. Participants in grades three to six were recruited from four mainstream primary schools in the greater Amsterdam region in the Netherlands. All schools belonged to one parent organization, with a single board involved in the management of 22 schools. Four schools were chosen based on the presence of participants with a broad range of socioeconomic statuses (SESs), ranging from low to high. The SES of the participants was evaluated according to the mean income and educational levels of the individuals living in the school’s catchment area (Status scores, 2016; Central Bureau for Statistics [CBS], 2017a). By including a roughly equivalent number of participants from low, moderate, and high SES families, we controlled for the SES differences that may have otherwise interfered with our main outcomes. Participation was voluntary. All parents or caregivers (referred to as caregivers in the rest of this paper) were informed that no personal information would be obtained and all data were assembled and analyzed at group level. The caregivers gave written permission for their child to participate. The Ethics Committee of the Faculty of Behavioral and Movement Sciences of the Vrije Universiteit Amsterdam approved the study protocol.

A fixed battery of 13 neuropsychological tests was administered to participants. This battery took approximately 60 min to administer. Moreover, teachers and parents of the participants in the study were asked to fill out an online questionnaire including a measure of teacher-perceived self-regulation (see van Tetering and Jolles, 2017). Finally, information on nationally used school achievement tests was provided by the participants’ school.

#### Level of Parental Education

Caregivers received an e-mail with login details to the online questionnaire. Both caregivers were asked to indicate their highest level of education on a nine-point scale (0 = no finished education to 9 = post university). This classification is based on the International Standard Classification of Education (Singh, 2010). This is a statistical framework on maintained education that is suitable for assembling, compiling, and presenting education statistics, both within individual countries and internationally. In the present study, LPE was dichotomized into two levels: moderate-to-low LPE (i.e., vocational training or lower) and high LPE (i.e., higher than vocational training). Dichotomization of LPE was based on the frequency distribution of LPE in order to create two groups with comparable sample sizes.

#### Inclusion of Participants

Level of parental education estimates and a fully answered teacher-report questionnaire related to self-regulation were essential for inclusion in the study. Participants who accelerated or delayed a class were excluded (*n* = 87). This was done in order to have a relatively homogeneous sample with typically developing participants and to prevent overlap in ages between grades. The use of sharp age-boards between grades is according to the October-norm. Inclusion of participants was therefore based on their date of birth. To exemplify the third grade: all participants born before 1 October 2004 or after 1 October 2005 were excluded (Rijksoverheid, 2017). Supplementary Figure 1 gives an overview of the inclusion and exclusion of participants. *n =* 211 subjects were eligible and participated in the study.

#### Design: Investigating Four Research Questions in Four Different Study Samples

There were four research questions; three of them pertained to the school achievement tests, namely (1) mathematical ability, (2) reading comprehension ability, and (3) spelling ability. The fourth question was related to the investigation of LPE differences. Because there were some missing data for each of these variables, four study samples were drawn in order to have as many subjects as possible for the investigation of the four research questions. Subject numbers were *n* = 201 for the investigation of mathematical ability, *n* = 202 for reading comprehension, *n* = 201 for spelling ability, and *n* = 211 for LPE differences.

#### Group Comparison: The Highest Versus the Lowest Tertile of School Performance

In the first three study samples a group comparison was done of participants with high and low school achievement. This approach was used to increase the statistical power to detect an effect by focusing exclusively on those participants who are highly representative of a specific skill, as explained by Preacher et al. (2005). This approach was chosen because the focus of the study was on individuals with high and low school achievement, and not on individuals with scores close to the mean (Pletti et al., 2017). In accordance with this design, individuals with high and low performance on each of the school achievement tests were determined for each grade based on the frequency distribution of the achievement test score. The highest tertile (33% of participants with the highest school achievement test scores of their grade) were defined as having high school achievement, and participants with the lowest 33% (lowest tertile) were defined as having low school achievement. Individuals with middle levels of school achievement (i.e., performance >33 and <66% of the cumulative frequency of their grade) were not used in the comparison (see Supplementary Figure 1).

#### Matched Case–Control Design

As a second approach, a matched case–control design was used in which each participant with high school achievement was individually matched to a participant with low achievement based on their age, grade, sex, school, and LPE. This is an often-used method in clinical epidemiological studies (see for example Bouter and van Dongen, 2006; Vandenbroucke and Hofman, 2010). In the present study, this method was chosen to control for sex, LPE, and school differences between the participants with high and low school achievement. Findings of previous studies suggest that these factors could interfere with our main outcome of interest (i.e., self-regulation skills). For instance, previous studies show that cognitive abilities including self-regulatory skills generally improve with age (see for example Ursache et al., 2012). Moreover, van Tetering and Jolles (2017) reported sex differences on various self-regulatory skills as perceived by teachers. They also reported that LPE influenced self-regulatory skills as perceived by teachers (van Tetering and Jolles, 2017). Finally, differences in the development of cognitive abilities between schools can be considered. This is because each school has its own population of students. Students generally go to a school nearby their home (Central Bureau for Statistics [CBS], 2017b). Accordingly, children living in higher socio-economic neighborhoods go to the same schools, even as children growing up in lower socio-economic neighborhoods. As socio-economical background may influence cognitive development (see Sarsour et al., 2011; Trzaskowski et al., 2014; Lawson et al., 2017), it is needed to control for school differences. Otherwise school differences could interfere with our main outcome. For these reasons, children with high and low school achievement were matched based on their age, grade, sex, school, and LPE. Supplementary Figure 1 gives an overview of the matching procedure. This procedure has been used in numerous of other studies (e.g., see Preacher et al., 2005; Pletti et al., 2017; Schlier et al., 2017).

The final number of subjects in the first three study samples was *n* = 92 for the investigation of mathematical ability, *n* = 90 for reading comprehension, and *n* = 70 for spelling ability. The demographic characteristics of the three samples are shown in **Table 1**.

**Table 1:** Demographic variables of the case–control group for each grade.

	Grades			
	3	4	5	6
**Mathematics**				
High/low school performance (*n*)	7/7	10/10	14/14	15/15
Boys/girls (*n*)	2/12	8/12	16/12	16/14
Age (*M, SE*)	8.8 (0.1)	10.2 (0.1)	11.1 (0.1)	12.0 (0.1)
Minimum–maximum age	8.6–9.2	9.8–10.5	10.6–11.5	11.6–12.5
LPE (*M, SE*)	5.9 (0.3)	6.3 (0.3)	6.2 (0.2)	6.2 (0.2)
Low-to-moderate/high LPE (*n*)	10/4	8/12	18/10	16/14
**Spelling**				
High/low school performance (*n*)	6/6	7/7	14/14	8/8
% Girls	6/6	6/8	18/10	6/10
Age (*M, SE*)	9.0 (0.1)	10.0 (0.1)	11.0 (0.1)	12.0 (0.1)
Minimum–maximum age	8.6–9.3	9.7–10.4	10.6–11.5	11.6–12.5
LPE (*M, SE*)	6.7 (0.2)	6.0 (0.4)	6.2 (0.3)	6.0 (0.4)
Low-to-moderate/high LPE (*n*)	6/6	10/4	15/13	10/6
**Reading comprehension**				
High/low school performance (*n*)	7/7	9/9	15/15	14/14
% Girls	2/12	8/10	26/4	16/12
Age (*M, SE*)	9.0 (0.1)	10.0 (0.1)	11.0 (0.1)	12.00 (0.1)
Minimum–maximum age	8.6–9.3	9.7–10.5	10.6–11.5	11.6–12.5
LPE (*M, SE*)	6.4 (0.3)	5.9 (0.4)	5.9 (0.3)	6.3 (0.3)
Low-to-moderate/high LPE (*n*)	6/8	10/8	18/12	16/12

#### The Fourth Study Sample: Matching High to Moderate-to-Low LPE

A similar matching case–control procedure was used to select the fourth study sample. This was devised to investigate the importance of LPE in self-regulation. Participants with high LPE were matched to participants with moderate-to-low LPE. The final number of subjects for LPE differences was *n* = 164. Demographic characteristics of the fourth study sample are also presented in **Table 1**.

### Instrument

#### Measurements of School Achievement: The CITO Tests

Mathematics, spelling, and reading comprehension ability were assessed with three nationally used paper-and-pencil achievement tests that are standardized and nationally norm-referenced. These tests have been developed by the Dutch Standard Central Institute for Test Development [i.e., in Dutch: Centraal Instituut voor Toetsontwikkeling (CITO)]. The three CITO tests are the CITO mathematics test (Janssen et al., 2010), the CITO spelling test (de Wijs et al., 2010; Mols and Kamphuis, 2012), and the CITO reading comprehension test (Feenstra et al., 2010; Weekers et al., 2011). The internal consistencies of the three tests are used as a measure of reliability, and are reported to be high (i.e., internal consistency of CITO mathematics grades 3–6 ranges from 0.91 to 0.97, see Janssen et al., 2010; that of CITO spelling grades 3–6 ranges from 0.87 to 0.94, see de Wijs et al., 2010; Mols and Kamphuis, 2012; and that of CITO reading comprehension grades 3–6 ranges from 0.83 and 0.93, see Feenstra et al., 2010; Weekers et al., 2011). The validity of the CITO tests is considered to be high since (1) calibration research showed that the differences in participant performances on all three CITO tests could be explained by one unidimensional concept, (2) it appeared that all three of the CITO test performances were highly correlated with similar abilities that were measured with other subparts of the CITO tests, and (3) participants’ performances on a CITO test of a particular domain was predictive for performance on the following CITO test of that domain.

In the present study, the “skill-scores” (i.e., translated from the Dutch “vaardigheidscores”) were used as a measure for cognitive performance. The skill-score is calculated for each CITO- test, which is a compilation-score based on the various abilities assessed in each test. These scores are known to improve over the years and are useful in monitoring the progression on each of the CITO tests (de Wijs et al., 2010; Feenstra et al., 2010; Janssen et al., 2010; Weekers et al., 2011; Mols and Kamphuis, 2012). There are two different tests for each grade, one regularly administered halfway through the year (January) and one around June. In this study, we used the CITO test results obtained in January 2014.

#### Mathematical Abilities

The Dutch standard CITO mathematics test was used to assess various mathematical abilities (Janssen et al., 2010). Participants fill out their answers on a piece of paper. The test took 40 min to administer in grades 3, 4, and 5, and 45 min in grade 6. In grades 3 to 6, the following math skills are covered in the test: (a) number and number relations; (b) addition and subtraction; (c) multiplication and division; (d) measuring (e.g., weights, length, surface, time). From grade 4, (e) percentages and fractions are also covered.

#### Spelling Abilities

The Dutch standard CITO spelling test was used to assess implicit spelling abilities (de Wijs et al., 2010; Mols and Kamphuis, 2012). The test took 30 min to administer. Spelling ability is tested by (1) dictated words, (2) dictated sentences, and (3) questions where participants have to indicate the sentence with the wrongly spelled word (in bold case) out of four different sentences.

#### Reading Comprehension Abilities

The Dutch standard CITO reading comprehension test was used to assess reading comprehension abilities (Feenstra et al., 2010; Weekers et al., 2011). The test took approximately 40 min to administer. Reading comprehension is tested by (1) questions related to the facts and events in a text, and (2) filling in words that fit in a short textual passage.

#### Measurement of Teacher-Perceived Self-Regulation Skills: The AEFI

The AEFI (Van der Elst et al., 2012) was originally developed to measure self-regulation and associated executive functions by means of a short self-reported questionnaire. It has also recently been used as a teacher- and parent-report questionnaire (Van der Elst et al., 2012; van Batenburg-Eddes and Jolles, 2013; Baars et al., 2015; van Tetering and Jolles, 2017). It consists of 13 items that represent three dimensions of executive functioning: (1) Attention (three items); (2) Planning and initiative taking (five items); and (3) Self-control and self-monitoring (five items) (see also Van der Elst et al., 2012). The AEFI took between 5 and 10 min to complete. The observer-report version of the AEFI used in the present study had some minor differences in the examples that were given to explain the questions. This was done in order to make the questions age appropriate. The items and examples were identical to those used by van Tetering and Jolles (2017). The teachers were asked to indicate how well each item described the child by endorsing one of three responses on a three-point Likert scale: “1 = not true,” “2 = partly true,” “3 = true.” Items 1, 4, 5, 6, 7, 8, 11, 12, and 13 were reverse coded, and the total score of all items was calculated so that higher scores were indicative of better self-regulation and executive functions.

The validity of the AEFI was previously evaluated in a large study of adolescents aged 15–18 and has been reported to be adequate (Van der Elst et al., 2012). The reliability and internal consistency of the AEFI when used as an observer questionnaire were extensively investigated in van Tetering and Jolles (2017). To ensure that the reliability and the internal consistency of the AEFI were acceptable in this study, we re-examined the four samples in the present study. The results were essentially the same in each study sample: the Cronbach’s alphas ranged between 0.72 and 0.79 on the attention scale, between 0.63 and 0.70 on the self-control and self-monitoring scale, and between 0.77 and 0.83 on the planning and initiative taking scale. In addition, the corrected item-scale correlations (i.e., the correlations between items and scale scores that did not include the items being evaluated) were calculated to be within the guideline of ≥0.30 (Ferketich, 1991). For shorter scales, the corrected item-scale values provide a better index of internal consistency and reliability than Cronbach’s alpha, because Cronbach’s alpha values are not only a function of the height of the inter-correlations between the items of a scale, but are also a function of the number of items on that scale (Clark and Watson, 1995). Again, the inter-item correlations in all four study samples were essentially the same. It was thus concluded that the reliability and the internal consistency of the AEFI used in the present study were adequate. The Cronbach’s alphas and the inter-item correlations of the study samples are presented in Supplementary Table 1.

### Statistical Analysis

Analyses were performed in IBM Statistics 23. The depended variable was normally distributed for each category of the independent variable (i.e., investigated by visual inspection of the histograms and skewness < 3, kurtosis < 10; Kline, 2005) and the assumption of equal variances (Levine’s test *p* > 0.05) was approved. *p*-Values less than or equal to 0.05 were considered statistically significant.

Differences in teacher-perceived self-regulation (dependent variable: total score AEFI and three AEFI scales: attention, planning and initiative taking, self-control and self-monitoring) were assessed between participants with high and low-to-moderate LPE (independent variable) using one-way analyses of variance (ANOVAs). Differences in teacher-perceived self-regulation (dependent variable: total score AEFI and three AEFI scales: attention, planning and initiative taking, self-control and self-monitoring) between participants with high and low school achievement (independent variable) were assessed according to a similar procedure.

## Results

### Self-Regulation of Participants With High LPE Versus Moderate-to-Low LPE

There were significant differences in teacher-perceived self-regulation between students from high versus moderate-to-low LPE families in favor of the high LPE students. The findings were statistically different for attention [*F*(1,162) = 4.41, *p* < 0.04, *d* = 0.33], for self-control and self-monitoring [*F*(1,162) = 4.44, *p* < 0.04, *d* = 0.33], and for the total AEFI score [*F*(1,162) = 5.24, *p* = 0.02, *d* = 0.36]. Means, standard errors, effect sizes, and results of the analyses are shown in **Table 2**.

**Table 2:** Level of parental education (LPE) differences on the AEFI scales.

	LPE					
	Low-to-moderate		High			
	*M*	*SE*	*M*	*SE*	Cohen’s *d*	*p*-Value
Attention	3.6	0.2	4.2	0.2	0.33	0.037^∗^
Planning and initiative taking	5.7	0.3	6.4	0.3	0.26	0.102
Self-control and self-monitoring	7.3	0.3	8.1	0.3	0.33	0.037^∗^
Total AEFI score	16.6	0.7	18.7	0.7	0.36	0.023^∗^

*^∗^*p* < 0.05.*

### Self-Regulation in Relation to Mathematics Achievement

There were statistically significant differences between teacher-perceived self-regulation of participants with high CITO mathematics test achievement in comparison to that of participants with low achievement. This was found for attention [*F*(1,90) = 16.21, *p* < 0.01, *d* = 0.84], for planning and initiative taking [*F*(1,90) = 55.82, *p* < 0.01, *d* = 1.56], for self-control and self-monitoring [*F*(1,90) = 8.65, *p* < 0.01, *d* = 0.61], and for total AEFI score [*F*(1,90) = 38,73, *p* < 0.01, *d* = 1.30]. Participants with high CITO mathematics test achievement had higher self-regulation skills than participants with moderate-to-low achievement.

### Self-Regulation in Relation to Spelling Achievement

There were statistically significant differences between teacher-perceived self-regulation of participants with high CITO spelling test achievement in comparison to that of participants with low test achievement. This was found for attention [*F*(1,68) = 9.00, *p* < 0.01, *d* = 0.72], for planning and initiative taking [*F*(1,68) = 10.81, *p* < 0.01, *d* = 0.79], and for total AEFI score [*F*(1,68) = 11.91, *p* < 0.01, *d* = 0.82]. Participants with high CITO spelling test achievement had higher self-regulation skills than children with low spelling test achievement. The difference in teachers’ evaluation on the scale self-control and self-monitoring approaches significance [*F*(1,68) = 2.95, *p* = 0.09, *d* = 0.41].

### Self-Regulation in Relation to Reading Comprehension Achievement

There were statistically significant differences between teacher-perceived self-regulation of participants with high CITO reading comprehension test achievement in comparison to that of participants with low test achievement. This was found for attention [*F*(1,88) = 10.49, *p* < 0.01, *d* = 0.68], for planning and initiative taking [*F*(1,88) = 25.09, *p* < 0.01, *d* = 1.06], for self-control and self-monitoring [*F*(1,88) = 8.16, *p* < 0.01, *d* = 0.60], and for the total AEFI score [*F*(1,88) = 24.05, *p* < 0.01, *d* = 1.03]. Participants with high CITO reading comprehension test achievement had higher self-regulation skills than in participants with low test achievement. Means, standard errors, effect sizes, and results of the analyses for each CITO test are shown in **Table 3**.

**Table 3:** Results children with high and low results on three CITO achievement tests.

	School achievement					
	Low		High		Cohen’s *d*	*p*-Values
CITO	*M*	*SE*	*M*	*SE*		
**Mathematics test**						
Attention	3.3	0.3	4.7	0.2	0.84	<0.001^∗^
Planning and initiative taking	4.3	0.3	7.6	0.3	1.56	<0.001^∗^
Self-control and self-monitoring	7.1	0.4	8.4	0.3	0.61	0.004^∗^
Total AEFI score	14.7	0.7	20.8	0.6	1.30	<0.001^∗^
**Spelling test**						
Attention	3.1	0.3	4.3	0.3	0.72	0.004^∗^
Planning and initiative taking	5.0	0.5	7.1	0.4	0.79	0.002^∗^
Self-control and self-monitoring	7.1	0.4	8.0	0.3	0.41	0.090
Total AEFI score	15.2	<1.0	19.3	0.3	0.82	0.001^∗^
**Reading comprehension test**						
Attention	3.4	0.3	4.6	0.3	0.68	0.002^∗^
Planning and initiative taking	4.5	0.4	7.3	0.4	1.16	<0.001^∗^
Self-control and self-monitoring	7.4	0.3	8.6	0.3	0.60	0.005^∗^
Total AEFI score	15.3	0.8	20.4	0.7	1.03	<0.001^∗^

*^∗^*p*-value < 0.05.*

## Discussion

The first study in this paper investigated whether children with high and low academic achievement differ in their levels of self-regulation as perceived by teachers. It was then evaluated whether there are differences in the self-regulation as perceived by teachers of children with high and moderate-to-low LPE. As hypothesized, teachers evaluated self-regulation as lower in students with low school achievement compared to children who performed well at school. This applies to performance in the three cognitive domains tested, i.e., mathematics, spelling, and reading comprehension. In addition, teachers reported lower levels of self-regulation for children growing up in moderate-to-low LPE families in comparison to children growing up in high LPE families. This is also in agreement with our expectations. The findings offer strong support for the notion that teachers perceive the self-regulation of students to be highly important for school achievement. The findings are especially strong since we applied a strict case–control design. This prevents the interference of factors such as sex, age, and LPE.

The findings are relevant for educational practice in that they underscore the importance of self-regulation for achievement in an educational setting, as suggested by our earlier studies (Baars et al., 2015; Nije Bijvank et al., 2017; van Tetering and Jolles, 2017) and by other researchers (Kent et al., 2014; Dekker et al., 2017). In the present study, the teachers reported that attention and planning and initiative taking are important in mathematics, spelling, and reading comprehension. Teachers reported the level of these skills to be higher in children with high school achievements than in children with low school achievements in these cognitive domains. Furthermore, teachers reported that self-control and self-monitoring were additionally important in mathematics and reading comprehension, as teachers reported the level of these skills to be higher in children with high mathematics and reading comprehension achievement than in children with lower achievements.

These findings support those of previous studies on the importance of self-regulation to school achievement (e.g., Kent et al., 2014; Gerst et al., 2015; Dekker et al., 2017). For instance, our findings confirm those of Gerst et al. (2015), who reported on the importance of planning abilities to mathematics and reading comprehension achievements, and the findings of Kent et al. (2014), who reported on the importance of attention for spelling achievement. Furthermore, Dekker et al. (2017) reported that teacher evaluations about the students’ self-regulation added unique predictive value next to behavioral measures to spelling achievement. Our study further substantiates their findings: they suggest that teacher evaluations are highly relevant when assessing the self-regulation of students as important for spelling, mathematic, and reading comprehension achievements.

Teacher evaluations were assessed with the aid of the AEFI, a short observer-report questionnaire (Van der Elst et al., 2012; van Tetering and Jolles, 2017). Our findings extend the findings of other studies using performance tests (e.g., Locascio et al., 2010; Best et al., 2011; Diamond, 2013; Friso-van den Bos et al., 2013; Arrington et al., 2014; Cragg and Gilmore, 2014; Gerst et al., 2015; Ten Eycke and Dewey, 2016; Dekker et al., 2017; Schwaighofer et al., 2017). This is important because these studies validate the findings of studies conducted using an observer-report: the judgment of the teacher is therefore of value in the school environment as teachers evaluate their students on a day-to-day basis. Our findings confirm the notion that teachers are able to differentiate between the self-regulation of children with higher and lower levels of school achievements.

There are several explanations for the relationship between the self-regulation skills of the student and their school achievement. The first explanation is that self-regulation has a direct effect on the student’s performance in achievement tests. Children with lower levels of self-regulation may have difficulties paying attention to a test for a long period of time (the so-called ‘sustained attention’; Anderson, 2002; Diamond, 2013). These children may also have difficulties in planning and prioritizing the smaller steps needed to solve an assignment or with suppressing irrelevant impulses and information while taking a test (Diamond, 2013; Gerst et al., 2015). Lower self-regulation skills could therefore negatively affect test performance directly. A second possible explanation for the relationship between self-regulation and school achievement is that children who are better at planning their homework and at paying attention in the classroom have an advantage over children with lower levels of these executive functions because it is easier for them to gain knowledge and benefit from earlier learning experiences in general (Anderson, 2002; Diamond, 2013). These children may have gained more knowledge at home or at school and are therefore better at identifying relationships both within subjects and between subjects. They may also be better in adjusting their behavior toward new situations because they have more experience with such activities (Anderson, 2002; Gerst et al., 2015; Jolles, 2016). As a result, children with better self-regulation skills have an advantage that could help them to acquire more knowledge and experience and to obtain higher educational levels later in life, as has also been suggested by previous researchers (e.g., Locascio et al., 2010; Best et al., 2011; Diamond, 2013; Friso-van den Bos et al., 2013; Arrington et al., 2014; Cragg and Gilmore, 2014; Gerst et al., 2015; Dekker et al., 2017; Schwaighofer et al., 2017). These previous findings and our results suggest that by improving self-regulation, school achievement levels could improve as well. Moreover, programs aimed at stimulating the development of self-regulation may not only foster children’s learning abilities but they could also enhance the classroom environment, by reducing classroom stress while improving students’ ability to pay attention and to monitor their own learning (Ursache et al., 2012).

This is a topic which will be the focus of a forthcoming study in our department.

It appears that the educational levels that individuals achieve strongly determines the position that individuals gain in society (see for example Cutler and Lleras-Muney, 2006; Laird et al., 2007; Meng et al., 2009). It determines the kind of job that individuals obtain later in life, their salary and responsibilities, and eventually their SES (e.g., Laird et al., 2007; Meng et al., 2009; Organization for Economic Co-operation and Development [OECD], 2011). Educational levels have also been related to mental and physical health (e.g., Gottfredson and Deary, 2004; Organization for Economic Co-operation and Development [OECD], 2011). It is therefore highly important to gain knowledge about factors that affect self-regulation and thus school achievement, such as those investigated in the present study. Insight into these factors can offer the opportunity to provide personalized support to improve these factors and academic performance accordingly.

The results of the present study suggest that the LPE of the family in which children grow up is a factor that contributes to individual differences in self-regulation as perceived by teachers. Well-educated parents tend to create a more intellectually stimulating environment for their children than less well-educated parents (e.g., Hoff, 2003). A stimulating environment affects the complexity of language used, the books read, the availability of playing materials, the level of ambitions that parents have for their developing child, as well as school attendance and general cognitive development (Ganzach, 2000; Hoff et al., 2002; Carr and Pike, 2012; Kautz et al., 2014). The results of the present study give strong support for our view that these factors positively stimulate the development of self-regulation and thereby contribute to better school achievement. This implies that children from less educated families should receive special attention in the development of self-regulation. However, our findings with respect to LPE differences in self-regulation should be interpreted with some caution. From a neuropsychological perspective, we investigated LPE differences on separate and distinctive cognitive abilities administered in one task. It was expected that LPE may selectively affect some of the outcome measures and not others. It is of special relevance for future research to replicate our findings in a larger study to determine whether the effects of LPE that were found in this study remain significant. Moreover, future research should use a more specific measure of LPE. LPE was dichotomized in our study, which was the best option considering the sample size. Nevertheless, this procedure resulted in two groups which were both characterized by a quite broad range of educational levels. This may have weakened our results because clear differences in the degree to which parents create an intellectually stimulating environment for their children can be expected between less well-educated parents and those who obtained better (moderate) educational levels. Future research should therefore focus upon a new measure of LPE which is more sensitive to subtle differences in the level of education obtained by the parents. It is also important to take into account that the estimated level of education of some caregivers was lower than the actual level obtained due to post-initial education or in-corporate training or courses. Future research should therefore take occupation into consideration. A more elaborate and sensitive estimation of LPE will give a better approximation of the intellectual climate within a family.

In order to interpret the results presented here correctly, three points need to be discussed. First, there is a possibility that the evaluations of teachers are based on their foreknowledge about a student’s school achievement. For example, teachers could generalize the low levels of school achievement of children to other skills, such as self-regulation (“this child does not perform well in school achievement tests, so he has an inferior self-regulation”). The same could be said for the relationship between LPE and teacher-perceived self-regulation: evaluations could be influenced by prior knowledge of the child’s home environment. This would indicate that the reflections of teachers do not necessarily represent the actual performance of a student. Rather, they represent the expectations of teachers shaped by prior knowledge about the students’ home environment. If teacher-evaluations are influenced by prior knowledge, it is considerable that grades given by teachers (rather than scores on national performance tests) are especially influenced by their impression of the student. For instance, teachers may give lower grades to students when they believe that students have poor self-regulation skills. It is therefore to be expected that teacher-perceived self-regulation is even stronger associated to school grades than to national performance tests. If this indeed is the case, then it is needed to create awareness to teachers regarding this phenomenon to prevent that children with poor school achievement and lower LPE are more negatively evaluated on broader domains. Future research should therefore examine the relation between self-regulation and school grades. Notably, findings from behavioral and observational studies on the relationship between self-regulation and academic achievements confirm that children with lower levels of school achievements have lower levels of self-regulation than children with higher levels of school achievements (e.g., Locascio et al., 2010; Best et al., 2011; Diamond, 2013; Friso-van den Bos et al., 2013; Arrington et al., 2014; Cragg and Gilmore, 2014; Gerst et al., 2015; Dekker et al., 2017; Schwaighofer et al., 2017). The same accounts for earlier studies on the link between LPE and self-regulation in children (Ganzach, 2000; Ardila et al., 2005; Meijs et al., 2009; Evans et al., 2010; Carr and Pike, 2012; Kautz et al., 2014; Gerst et al., 2015; Rindermann and Baumeister, 2015; van Tetering and Jolles, 2017). These studies confirm that sex and LPE are relevant factors which contribute to individual variations in self-regulation.

A second point to discuss is the use of the extreme groups approach (in which the third tertile was compared to the first tertile) in order to determine whether children showed high or low school performances. This approach has been used in many other studies (e.g., Preacher et al., 2005; Pletti et al., 2017; Schlier et al., 2017; Taruffi et al., 2017). Advantage of this approach is that it increases efficiency because it ensures similar numbers of cases and controls in confounder strata (Pearce, 2016). Another advantage is that the use of the extreme groups approach fits well into the daily practices of the classroom. For instance, learning new information is straightforward for some children, while other children experience difficulties. Our study showed that teachers observe differences in the self-regulation of these children. An often-reported problem using the extreme groups approach is that the scores on the extreme are vulnerable for regression toward the mean. This could affect the test–retest reliability of the studies but this cannot be considered as a potential problem in our study since two-thirds of the children were included, and only one-third of the children were excluded (e.g., the children with average CITO achievements). Moreover, we performed *post hoc* analyses to compare the teacher-perceived self-regulation of children that obtained moderate CITO test achievements with those of children that obtained low and high achievements. These *post hoc* analyses were performed on the hypothesis that finding more subtle differences in teacher-evaluated self-regulation between children with smaller differences in school achievements will provide additional support for the importance of self-regulation in school achievements. Results revealed that there were clear differences between children with low versus moderate, and moderate versus high CITO test achievements. These findings therefore suggest that even if data of our extreme groups were vulnerable for regression toward the mean, our findings are still relevant since self-regulatory skills even differ between children with low versus moderate school achievement, and moderate versus high achievement (for results, see Supplementary Table 2).

Final point to discuss is the importance of taking the developmental character of self-regulation into consideration while studying this ability in the period of late childhood and early adolescence (i.e., at the age of 8–12 years as in this study) (Jahromi and Stifter, 2008; Ursache et al., 2012; van Tetering and Jolles, 2017). Earlier studies reported that self-regulation continues to improve over the teenage years well into the mid-20s (Diamond, 2013). Strength of our study is therefore that we matched each student on age to make a fair comparison with respect to their level of self-regulation. Yet, because of the design of our study we cannot elaborate on the developmental character of teacher-perceived self-regulation.

## Conclusion

This study discussed the importance of teacher-evaluated self-regulation in school achievements in children aged 8–12. The results of this study also show that LPE – and thus factors related to upbringing – contribute to individual differences in the developmental trajectories of self-regulation. The results suggest that it is important that children gain experience with activities that stimulate the development of self-regulation early in life, since self-regulation is important for achievements at school. A finding with applied potential from our study is that teacher evaluations regarding the students’ self-regulation can be considered of value in school practice. Instruments which focus upon executive functioning, such as the AEFI, thus have potency for use in educational settings.

## Author Contributions

MT made substantial contributions to the conception and design of the work, to the analysis and interpretation of data, and to drafting the work. RG made substantial contributions to the design of the work, to the interpretation of data, and to revising the work critically for important intellectual content. JJ made substantial contributions to the conception or design of the work, to the acquisition and interpretation of data for the work, and to revising the work critically for important intellectual content. All the authors gave the final approval of the version to be published and agreed to be accountable for all aspects of the work in ensuring that questions related to the accuracy or integrity of any part of the work are appropriately investigated and resolved.

## Conflict of Interest Statement

The authors declare that the research was conducted in the absence of any commercial or financial relationships that could be construed as a potential conflict of interest.
