# Supplementary material for: Teacher-Evaluated Self-Regulation Is Related to School Achievement and Influenced by Parental Education in Schoolchildren Aged 8–12: A Case–Control Study
Source: Front Psychol. 2018 Apr 4;9:438. doi: 10.3389/fpsyg.2018.00438 (PMC5893787; doi:10.3389/fpsyg.2018.00438)
Supplement: Supplementary file 1 [file Table_1.docx]

Supplementary Material

**The school achievement of children aged 8-12 is related to teacher-perceived self-regulation and the level of parental education: A case-control study**

M.A.J. van Tetering*, R. de Groot & J. Jolles

*** Correspondence:** M.A.J. van Tetering: [m.a.j.van.tetering@vu.nl](mailto:m.a.j.van.tetering@vu.nl)

# Supplementary Tables

**Table 1.** Results of Reliability Statistics on the AEFI scales.

*Note.* AEFI = Amsterdam Executive Functioning Inventory; *r*is = corrected item–scale correlation.

|  | Mathematics  Sample |  | Spelling  Sample |  | Reading comprehension Sample |  | LPE Sample |  |
| --- | --- | --- | --- | --- | --- | --- | --- | --- |
| *AEFI item* | Cronbach’s Alpha | r_is_ | Cronbach’s Alpha | r_is_ | Cronbach’s Alpha | r_is_ | Cronbach’s Alpha | r_is_ |
| Attention | 0.78 |  | 0.72 |  | 0.75 |  | 0.79 |  |
| 5. He/she is not able to focus on the same topic for a long  period of time |  | 0.67 |  | 0.54 |  | 0.56 |  | 0.62 |
| 9. He/she is easily distracted |  | 0.62 |  | 0.58 |  | 0.67 |  | 0.70 |
| 12. His/Her thoughts easily wander |  | 0.57 |  | 0.49 |  | 0.52 |  | 0.60 |
| Self-control and Self-monitoring | 0.66 |  | 0.65 |  | 0.63 |  | 0.70 |  |
| 1. He/she often reacts too fast. He/she has done or said something before it is his/her turn. |  | 0.32 |  | 0.40 |  | 0.31 |  | 0.35 |
| 7. He/she often loses things |  | 0.64 |  | 0.68 |  | 0.56 |  | 0.67 |
| 8. It takes a lot of time for him/her to finish tasks |  | 0.22 |  | 0.12 |  | 0.27 |  | 0.22 |
| 11. He/she often forgets what he/she has done yesterday |  | 0.35 |  | 0.29 |  | 0.18 |  | 0.43 |
| 13. It is difficult for him/her to sit still |  | 0.62 |  | 0.62 |  | 0.65 |  | 0.66 |
| Planning & Initiative taking | 0.77 |  | 0.79 |  | 0.83 |  | 0.81 |  |
| 2. It is easy for him/her to come up with a different solution if he/she gets stuck when solving a problem |  | 0.67 |  | 0.70 |  | 0.77 |  | 0.70 |
| 3. He/she is well-organized. For example, he/she is good at planning what he/she needs to do during a day |  | 0.51 |  | 0.55 |  | 0.60 |  | 0.59 |
| 4. He/she is full of new ideas |  | 0.49 |  | 0.53 |  | 0.64 |  | 0.57 |
| 6. He/she is curious, he/she wants to know how things work. |  | 0.46 |  | 0.42 |  | 0.60 |  | 0.51 |
| 10. He/she can make fast decisions (e.g., in lesson) |  | 0.58 |  | 0.64 |  | 0.55 |  | 0.64 |
