# Supplementary material for: Teacher-Evaluated Self-Regulation Is Related to School Achievement and Influenced by Parental Education in Schoolchildren Aged 8–12: A Case–Control Study
Source: Front Psychol. 2018 Apr 4;9:438. doi: 10.3389/fpsyg.2018.00438 (PMC5893787; doi:10.3389/fpsyg.2018.00438)
Supplement: Supplementary file 2 [file Table_2.docx]

Supplementary Material

**The school achievement of children aged 8-12 is related to teacher-perceived self-regulation and the level of parental education: A case-control study**

M.A.J. van Tetering*, R. de Groot & J. Jolles

*** Correspondence:** M.A.J. van Tetering: [m.a.j.van.tetering@vu.nl](mailto:m.a.j.van.tetering@vu.nl)

# Supplementary Tables

**Table 2.** Mean and standard error of children with medium performance on CITO achievement tests and results of analyses comparing these children with low and high performing children.

|  |  | School Achievement |  |
| --- | --- | --- | --- |
|  | Medium | Medium vs. Low | Medium vs. High |
| *CITO* | M (SE) | *p*-values | *p*-values |
| Mathematics test (n = 46) |  |  |  |
| Attention | 3.7 (0.2) | 0.900 | 0.008* |
| Planning & Initiative taking | 6.0 (0.4) | 0.002* | 0.003* |
| Self-control & Self-monitoring | 7.7 (0.3) | 0.592 | 0.263 |
| Total AEFI score | 17.3 (0.8) | 0.038* | 0.003* |
| Spelling test (n = 75) |  |  |  |
| Attention | 4.0 (0.2) | 0.056 | >0.99 |
| Planning & Initiative taking | 6.0 (0.3) | 0.276 | 0.004* |
| Self-control & Self-monitoring | 7.8 (0.3) | 0.326 | >0.99 |
| Total AEFI score | 17.7 (0.7) | 0.071 | 0.416 |
| Reading Comprehension test (n = 61) |  |  |  |
| Attention | 3.8 (0.2) | 0.911 | 0.053 |
| Planning & Initiative taking | 6.2 (0.3) | 0.005* | 0.097 |
| Self-control & Self-monitoring | 7.4 (0.3) | >0.99 | 0.025* |
| Total AEFI score | 17.3 (0.7) | 0.150 | 0.010* |

*Note*. **p*-value < .05. Bonferroni correction was used to correct for multiple testing issues.
