# Supplementary material for: Teacher-Evaluated Self-Regulation Is Related to School Achievement and Influenced by Parental Education in Schoolchildren Aged 8–12: A Case–Control Study
Source: Front Psychol. 2018 Apr 4;9:438. doi: 10.3389/fpsyg.2018.00438 (PMC5893787; doi:10.3389/fpsyg.2018.00438)
Supplement: Supplementary file 3 [file Image_1.pdf]

Supplementary Material

The school achievement of children aged 8-12 is related to teacher-perceived self-regulation and the level of parental education: A case-control study

M.A.J. van Tetering*, R. de Groot & J. Jolles

*** Correspondence:** M.A.J. van Tetering: m.a.j.van.tetering@vu.nl

2 Supplementary Figure

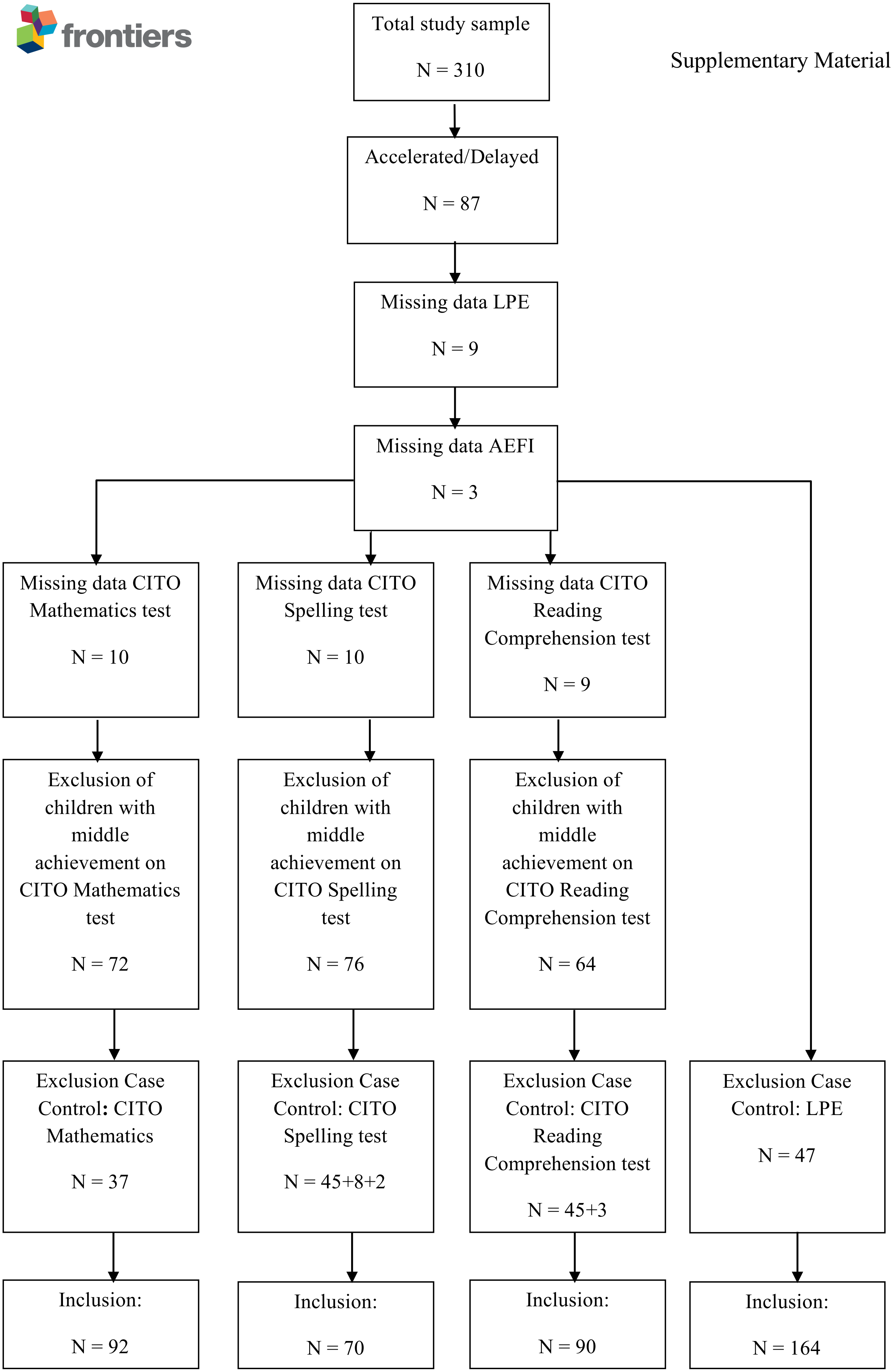

Supplementary Figure 1. Flowchart showing the selection of the study samples.
